# A healthy mistrust: how worldview relates to attitudes about breast cancer screening in a cross-sectional survey of low-income women

**DOI:** 10.1186/1475-9276-7-5

**Published:** 2008-01-31

**Authors:** Ann Carroll Klassen, Katherine C Smith, Salma Shariff-Marco, Hee-Soon Juon

**Affiliations:** 1 Department of Health, Behavior, and Society, Johns Hopkins Bloomberg School of Public Health, Baltimore, Maryland, USA; 2 Office of Preventive Oncology, National Cancer Institute, Bethesda, Maryland, USA

## Abstract

**Background:**

Perceived racial discrimination is one factor which may discourage ethnic minorities from using healthcare. However, existing research only partially explains why some persons do accept health promotion messages and use preventive care, while others do not. This analysis explores 1) the psychosocial characteristics of those, within disadvantaged groups, who identify their previous experiences as racially discriminatory, 2) the extent to which perceived racism is associated with broader perspectives on societal racism and powerlessness, and 3) how these views relate to disadvantaged groups' expectation of mistreatment in healthcare, feelings of mistrust, and motivation to use care.

**Methods:**

Using survey data from 576 African-American women, we explored the prevalence and predictors of beliefs and experiences related to social disengagement, racial discrimination, desired and actual racial concordance with medical providers, and fear of medical research. We then used both sociodemographic characteristics, and experiences and attitudes about disadvantage, to model respondents' scores on an index of personal motivation to receive breast cancer screening, measuring screening knowledge, rejection of fatalistic explanatory models of cancer, and belief in early detection, and in collaborative models of patient-provider responsibility.

**Results:**

Age was associated with lower motivation to screen, as were depressive symptoms, anomie, and fear of medical research. Motivation was low among those more comfortable with African-American providers, regardless of current provider race. However, greater awareness of societal racism positively predicted motivation, as did talking to others when experiencing discrimination. Talking was most useful for women with depressive symptoms.

**Conclusion:**

Supporting the Durkheimian concepts of both anomic and altruistic suicide, both disengagement (depression, anomie, vulnerability to victimization, and discomfort with non-Black physicians) as well as over-acceptance (low awareness of discrimination in society) predict poor health maintenance attitudes in disadvantaged women. Women who recognize their connection to other African-American women, and who talk about negative experiences, appear most motivated to protect their health.

## Background

Despite recent questions, most cancer control groups recommend annual mammography for women beginning at age 40[1]. In the United States, screening requires material resources such as access to care and means of payment, but also requires social and psychological resources to weigh the costs and benefits of early detection and treatment, and choose to enter the healthcare system. Research demonstrates that older, low-income African-American women, among other groups, are less likely to receive screening at recommended levels, even in situations where payment and access barriers are removed [2]. Suboptimal use of secondary prevention for breast cancer compounds the greater risk faced by African-American women from more aggressive tumor biology and younger onset of disease [3,4], and contributes to their excess breast cancer mortality compared to other ethnic groups in the U.S. [5]

Inequities in the secondary prevention of breast cancer have been traditionally framed in terms of barriers of access. Measurement of equitable distribution of preventive services such as mammography typically uses utilization as an endpoint; for example, by comparing rates of screening between groups [5]. However, in a critical analysis of the literature on access to healthcare, Dixon-Woods and colleagues [6] propose extending our conceptualization of equity in health care to include the more subjective concept of 'candidacy', defined as the patient's sense of legitimacy in using healthcare. Candidacy is the dynamic, "continually negotiated property of individuals, subject to multiple influences arising both from people and their social contexts and from macro-level influences on allocation of resources and configuration of services." Understanding how "vulnerabilities arise in relation to candidacy" may shed light on the roots of inequities in health and health care, by tying seemingly individual behaviors in utilization to socially patterned influences.

Research on cancer screening behavior has been dominated by fairly narrow fields of theory – individual psychological factors, or broad generalizations about the effect of social factors such as poverty, culture, gender or age. There is a need to use integrated approaches to examine how social factors shape behaviors, in order to reduce barriers to health-enhancing attitudes and behaviors, such as cancer screening.

One important area for such an integrated approach is in relation to how mistrust of the medical care system impacts use of care, especially for discretionary events such as preventive screening. The growing literature on health disparities acknowledges that the U.S. medical system has not always served those without power well [7]. Like all institutions, health care systems reflect the basic inequalities of the societies in which they function. Medical practitioners, as agents in a power asymmetry, may strive as individuals to reduce barriers for patients and build individual relationships that are positive, but may be powerless to fight the image of medical system as untrustworthy. Recent attention in the United States to the "Tuskegee Legacy" and the impact of this discussion on African-American attitudes towards medical care is but one of many social tensions being played out [8-10].

However, there is an equally strong argument that, especially among populations whose health is jeopardized across the life-course by many factors, access to and appropriate use of medical care is an important weapon for well-being. What are the best ways to understand barriers to preventive screening from both an individual and social perspective?

Research on societal-level influences on health, as well as studies of individual attitudes and beliefs, consistently find important differences in health behaviors between those in low resource environments compared to those in settings with better resources [11,12]; however, studies comparing health behaviors in advantaged versus disadvantaged groups often do not explore individual variation within social groups. Neither individual nor social approaches to studying health behaviors, by themselves, explain why, within low resource environments, some individuals exhibit hardiness – the ability to maintain health promoting attitudes and behaviors in the face of multiple barriers. In order to understand how the experience of social phenomena shapes individual differences in health attitudes, it is important to examine, from the individual's perspective, the interpretation of adverse social experiences, and the social resources used to combat them.

### Social resources and health

On both the macro and individual levels, theories of social resources share a common belief that such resources enhance health. On a societal level, theorists from Durkheim to Putnam have argued that social integration and connection to the larger society promotes health enhancing behaviors through social regulation and attachment, as well as providing the tools needed for achieving health, through social capital and shared social resources [13]. On an interpersonal level, social connectedness is also seen as health-promoting for most individuals, through both social role obligations and social support [14]. The relationship, however, between interpersonal social bonds and societal level roles is less clear.

When considering the role of social resources on health, it is possible to view societies as composed of nested social networks, working at various levels in a complementary way. On the other hand, it can be argued that affiliations within social groups come by definition only with exclusion of other groups, and that building of social capital for one group comes at the expense of the power of another [15]. From such a perspective, group identities and loyalties increase symbolic and material conflict between groups, leading not to social integration but disintegration.

### Disadvantage and discrimination: occurrence and measurement

Within societies, one interpretation of discrimination is as the result of struggles of groups to achieve occurring at the expense of other groups. For social researchers, as in the discussion of concepts such as stress, discrimination has emerged as multifaceted – it can be defined as the intent of the perpetrator, the interpretation by the recipient, or as the effect, or potential effect, of events and actions. Intent relies on the report of the perpetrator, and interpretation on the report by its recipient, while effect can be observed and measured by third parties.

As researchers, depending on our scientific philosophy, we must ask a related question as to whether our epistemology of discrimination is essentially based in a positivistic or more subjective, interpretivistic meaning and measurement [16]. Empirical studies of discrimination and health reflect this tension between externally defined, objective injustices and constructed or perceived discrimination.

The first is the measurement of incidents or processes that are defined as inherently discriminatory practices, or create de facto situations of discrimination, ranging from individual actions to discriminatory laws. These measures do not rely on the perceptions of the disadvantaged individual. Krieger [17] has labeled this type of discrimination as "indirect" because it measures result rather than intent or interpretation. The credibility and utility of such a consensus-based objectivistic approach is vulnerable to changing legal or cultural definitions of discrimination [18].

Perceived or reported discrimination, however, requires that individuals experience a situation in which they perceive themselves to be at a disadvantage compared to others, attribute that disadvantage to discrimination, and choose to disclose it to the questioner. Defining discrimination as an essentially subjective phenomenon means that if respondents report no discrimination, if they 1) do not feel they have received less, 2) attribute the difference to reasons other than their group membership, or 3) chose not to disclose their perceptions, then we must accept their authority in the interpretations of events. For example, in our previous use of questionnaire items on perceived discrimination in schooling, some older African Americans answered "No, I never experienced racial discrimination, because Blacks and Whites went to different schools," while other of their contemporaries answered affirmatively, reflecting the more common view that racially segregated schools were fundamentally discriminatory. Although a more positivist perspective would view these differing responses as problematic, a researcher whose goal was to understand "perceived discrimination" would focus on exploring these differences.

### Variation in reporting perceived racial discrimination

There is an international literature on ethnic and racial discrimination, focusing largely on discrimination toward either indigenous or immigrant non-white ethnic groups by economically or socially dominant white ethnic groups [19-25]. This literature shows wide variation in the discriminatory experiences reported, with variation depending on methodology and measures used, the characteristics of the discriminatory acts asked about (timing, type, setting, etc) and the sociodemographic characteristics of the respondents being questioned. This variation is also reflected in U.S. literature exploring racial discrimination among non-white groups. Historically, the majority of U.S. studies focus on the African-American experience; thus this literature provides the greatest evidence of the complexity of this issue.

Despite the prevalence of discriminatory practices in US society, national surveys [26] show that, for example, only 49% of Blacks report lifetime occurrence of major events of discrimination, while 71% report day-to-day discrimination as occurring "often" or "sometimes." Younger persons, and those with higher educational status are consistently more likely to report discrimination [26]. Adams and Dressler [27] found greater racism reported by African-Americans who had greater perceived personal influence, concluding "persons who see themselves as able to make changes are also more likely to perceive conditions that need changing." Others suggest that, for ethnic minorities, achieving greater social status clarifies discrimination as race- and not class-based [28]. These patterns are, on first glance, counter-intuitive, because we would anticipate that, if using an objective measure of negative experiences, those worse off would be most, rather than least, likely to report discrimination. Given that all African-Americans are subject to adverse conditions, it appears that those with greater personal resources are more likely to recognize, attribute, and willingly disclose discrimination. This paradox makes it difficult to separate, especially in cross-sectional measurement, the conditions accompanying or causing discrimination, the conditions facilitating its recognition, attribution and disclosure, and the conditions discrimination in turn truly produces. Measurement and framing effects further complicate comparisons across groups [29,30].

When considering older, African-American women living in poverty, who have lived for many years as members of not one but multiple groups subject to discrimination in the US – African-Americans, low income persons, and women – can we speculate on how labeling oneself a recipient of discrimination affects one's well-being? A conflict model would predict that this would build group consciousness, and move one from being a single victim to being a member of a larger struggle [31]. However, a social structural model would argue that recognizing one's distance from the majority society is not essentially an empowering experience, and quite the reverse, may serve to increase hopelessness and anomie.

Anomie has been conceptualized as a characteristic of societies [32] as well as individuals [33], and is a loss of orientation or norms, accompanied by, at the individual level, feelings of emptiness, meaninglessness and apathy. Merton [[33], p.230] described the "social typography of anomie" as "the structural places in American society... where the disjunction between the cultural values enjoining people to aim for certain goals and the patterned possibilities for living up to these values is at a maximum."

The relevance of this theory for understanding health-related behaviors such as preventive screening in disadvantaged populations is striking. Older low-income African-American women are often described to be at "quadruple jeopardy" of negative health or social outcomes due to four types of disadvantage – race, age, gender and social class disparities [34,35]. Yet from a sociological perspective these women are seldom described at being at risk for anomie, and disengagement from the social structures of their communities. Indeed, they are often stereotyped as singularly positive social resources within their weakened families, institutions, and communities [36]. How can we interpret this seeming hardiness against the negative effects of disadvantage, especially as it may relate to engagement in health-related preventive behaviors?

### Fight or flight: discrimination and health

Clark [37] uses a stress-coping model to argue that the psychological effects of discrimination can only occur when individuals recognize and respond to discrimination as a threat. Thus appraisal is a critical element in producing a range of responses, from maladaptive to adaptive. Some research suggests that the negative health consequences are greatest when discrimination is perceived and active steps are taken to combat it with limited resources, a psychological phenomenon labeled John Henryism [38]. Other researchers have found that not challenging situations of perceived discrimination was related to negative health outcomes ranging from increased blood pressure [39] to reduced access to health procedures [40]. It has been argued that both denial and overreaction to discrimination can be harmful; Krieger [17] offers evidence that both those who cannot identify any such experiences, and those who identify many, are more at risk for hypertension. However, Broman [41] found no relationship between racism and hypertension, and Jackson *et al*.'s [42] longitudinal study of reported racism found both positive and negative health effects. Overall, there is stronger evidence of a connection between discrimination and adverse mental health outcomes [43-45] such as depression [46] as well maladaptive behaviors including smoking [47], alcohol use [48], and violence [49].

### Disadvantage and discrimination in health care

In addition to the effects of discrimination in all aspects of life, discrimination specifically within the health care setting has warranted special focus as a proximal and powerful influence on health-related behaviors. Disadvantage in the quality of medical care received covers both process and outcome, and both are seen as distinct, yet interrelated.

A substantial body of evidence demonstrates that disadvantage exists in the receipt and quality of medical services, and that this directly influences the health of many groups in society [7,50]. Equally well studied is disadvantage in the medical care process – the quality of the interpersonal relationship between medical providers and patients, the satisfaction patients feel about their care [51], the trust they have in their individual provider and the system of care as a whole [52], and how that trust engenders social capital in the form of altruism, the willingness to give to others through acts such as organ and blood donation [53] or research participation [54]. Although evidence documenting disadvantage in the medical process is strong, causes and solutions are less clear cut. The study of patient-provider relationships has often focused on the interpersonal level [55,56], while studies of general dissatisfaction or disadvantage in medical care focus on system-level outcomes. However, as O'Malley [57] revealed, organizational characteristics can significantly influence patients' reports of trust, compassion, and communication, which are usually viewed as provider-level variables.

When studying racial and gender effects in medical care it is often argued that racial concordance between individual provider and patient can improve patient experiences for ethnic minority patients [58,59]. There is at least cross-sectional evidence that patients fearing discrimination are more likely to prefer same race providers [59,60], and that among patients preferring this, racial concordance leads to greater patient satisfaction [61]. However, there is also evidence that minority patients have fewer choices in medical care, and that minority providers have fewer choices of practice settings. Furthermore, even when there is racial concordance, if many minority physicians practice in, and many minority patients receive care from, lower resource medical environments, can cultural understanding, despite its importance, replace the material resources needed for high quality healthcare?

Although there are ample reasons for addressing historical inequalities of access in medical training and employment, the argument that a goal of patient-provider racial, cultural or gender concordance is, in and of itself, a solution to inequalities in health should be made cautiously. To do otherwise is to ignore the multiple pathways leading to these inequalities.

There is only a sparse literature, with varying measures, which directly examines the relationship between perceived racism, either globally or specifically within healthcare, and uptake of preventive services, especially specific to cancer screening. Structured reviews of the literature on disparities in colorectal [62], cervical [63], and breast [64] cancer found no studies examining the role of racism in relation to screening. Using 2001 national survey data, Blanchard [65] found mixed results, in that respondents believing they had been treated unfairly because of race were more likely to have optimal cancer screening, equally likely to report an exam within the past year, but were less likely to have optimal chronic disease screening, to follow doctors advice, and were more likely to delay care. Trivedi [66] found in the California Health Interview Survey that perceived discrimination in receipt of recent health care attributed to any reason (age, race, language, disability, insurance status, weight, income, gender or medical beliefs) was significantly predictive of lower rates of flu shots, hemoglobin A1c and cholesterol testing, and foot exam, but not prostate specific antigen (PSA) testing, or aspirin use.

#### Goals of this investigation

One legitimate focus of health disparities research to date has been to investigate the predictors of discrimination (whether perceived and self-reported, or externally assessed) with the important goal of identifying discrimination-producing situations, players and actions, and to suggest possible interventions to prevent its occurrence. However, an equally important avenue of research is to understand the effects of perceived discrimination on those who have experienced it, as a "harm reduction" strategy, to control the negative health consequences of discrimination.

In this analysis, we used data from a survey of older urban African-American women to explore the following questions.

1) What are the psychosocial characteristics of those, within disadvantaged groups, who perceive and report their own previous experiences as discriminatory?

2) To what extent is this perceived discrimination associated with broader perspectives on racism, power and powerlessness within society?

3) How do these society-level views relate to disadvantaged groups' expectation of mistreatment specifically within the medical care system, feelings of mistrust, and motivation to use care?

4) Does this suggest a possible pathway for how perceived discrimination influences attitudes towards cancer screening among this group of low-income urban African-American women? As a single example from one city, do findings contribute cross-sectional evidence towards either an empowering or disempowering role of perceived discrimination on the overall health and well-being of ethnic minorities within the US culture?

The model below illustrates one potential pathway for such influences. The actual experiences of racially-based mistreatment (depicted in brackets), are not directly observed or measured. Instead, they are interpreted by each respondent, and identified as discriminatory or not, based in part on her own social and psychological characteristics. This interpretation then may shape wider perspectives about race and power relationships in society, and the respondent's sense of her own power or powerlessness. This in turn may lead her to anticipate future negative events, such as mistreatment in the medical setting, and her likelihood of successfully combating them. (The pathways in this model are not unidirectional, but iterative across the lifetime; once formed, perspectives and beliefs will in turn shape a person's perceptions of new experiences of racism.) These views then contribute to a woman's motivation to accept health messages, including those related to breast cancer screening.

## Methods

### Population

Data used in these analyses come from a multi-year National Cancer Institute-funded study of breast cancer screening among African-American women in Baltimore, Maryland, a large US city. Methods and related findings have been previously published [2,67-69] and will be briefly described here. With the original purpose of evaluating the impact of a no-cost screening intervention within communities at risk for poor screening, we recruited all screening program participants age 45 and older residing in the 10 contiguous zipcodes of East Baltimore which served as the target catchment area of this program. This area, comprising roughly 40% of the City, contains both working class and extremely low income areas. We also recruited an age (± 5 years) and neighborhood-matched sample of participant-nominated friends and neighbors not attending the program. The 90-minute, in-home audiotaped interview was conducted by African-American female interviewers. During 1997 and 1998, we interviewed 576 women between the ages of 45 and 93, representing response rates of 83% and 86% from the clinic and nominated control sampling frames respectively. All participants provided written informed consent, and received $25 for participation. The study was approved by the Johns Hopkins Medical Institutions institutional review board.

The original case-control design was chosen to evaluate the impact of the screening program [2]. In addition, we geocoded all respondents by residential address; our comparison of respondents, using U.S. Census blockgroup-level data[70], to the sociodemographic characteristics of women in their neighborhoods supports analysis of the total group as a representative population of low and moderate income urban African-American women in East Baltimore, for questions not specifically related to the no-cost program [67-69].

### Measures used

In order to examine cancer and health in the context of older African-American women's lives, we developed our survey instrument using both open-ended questions eliciting each woman's views in her own words, as well as structured measures, chosen from our own or others' work in African-American and women's health.

### Independent measures: psychosocial measures

In these analyses, we use nine psychosocial covariates, including three sociodemographic measures: age, years of formal education, and self-reported household income, and two measures of physical and mental health status: each woman's rating of her health, and her responses on an abbreviated version of the CES-D [71] to measure depressive symptoms during the past week (Cronbach's Alpha = .82) [72]. In these analyses, we also incorporate four types of social connectedness: whether or not respondents currently worked, were homeowners, attended weekly religious activities, or were active in community events.

### Measures of beliefs and experiences

For these analyses, we used several measures to explore multiple aspects of the respondents' perspectives and experiences with power, both on a societal level and also within the health care system. We used two items which measured perspectives, conceptualized as shaped by but distinct from a woman's own experiences. A five item version of Scrole's [73] scale of anomie measured generalized hopelessness (Cronbach's Alpha = .74). We used thirteen items from Green's [74] Perceptions of Racism Scale to capture views on inequities facing African-American women in various areas of American society, including general racism (2 items), medical care (6), courts and government (2), job-seeking (1), education (1), and social class (1), measuring four levels of respondent agreement or disagreement with statements such as "Judges are harder on African-Americans than whites." (Cronbach's Alpha = .79).

Krieger *et al.*'s well-validated measures [75] were used to capture personal experience with, and response to, racial discrimination. Respondents were asked, when faced with unfair treatment, whether they generally "accepted it as a fact of life" or "tried to do something about it." Similarly, they were asked if they generally "talked to other people" about such experiences or "kept it to yourself." They were then asked if they had ever experienced "discrimination, been prevented from doing something, been hassled or made to feel inferior because of your race or color" in each of six types of settings (school, job hiring, work, housing, medical care, police/courts). We created a single dichotomous item indicating any experience of perceived racism, and two dichotomous possible types of reactions: talking to others, and trying to do something. To distinguish between measures, we label Green's Perceptions of Racism Scale as "Societal Racism" and responses to Krieger's measure of experiences of perceived discrimination "due to race or color" as "Reported (or perceived) Racism".

### Medical care experiences

We used two questions to create a four-category measure of whether or not the patient currently received medical care from a provider with whose race she felt comfortable. In a likert scale, we asked patients whether they strongly agreed, somewhat agreed, somewhat disagreed, or strongly disagreed with the statement, "I would be more comfortable seeing a doctor who was African-American than a doctor of another race." Elsewhere, we asked respondents whether their current primary provider was African-American. Women were grouped according to whether they had a primary provider who was African-American or not, and whether they agreed that they would be more comfortable with an African-American provider.

To specifically measure fear of deception in medical care, we asked the following: "Some people are afraid of being treated at big research hospitals like Johns Hopkins, because they are afraid they might be part of a research experiment without knowing it. Would you be concerned about that?"

### Outcome: positive attitude toward mammography

The focus of this investigation is attitudes and beliefs about the secondary prevention of cancer, rather than actual behaviors. In these data, consistent with existing literature, we have found that a woman's actual receipt of screening is influenced by many facilitators and barriers in addition to attitudes, including access to care, costs, and physician recommendation [2,67,68]. For these reasons, in this analysis, we chose to examine screening-related motivation, an important psychological component of health behavior in its own right, rather than the respondents' actual patterns of screening.

We operationalized our outcome variable as an index (appendix), summing respondents' answers to eleven questionnaire items regarding breast cancer and screening. We theorize that women with high scores on this index had an understanding of breast cancer and mammography compatible with cancer control strategies promulgated by the medical community, as well as willingness to use the majority culture medical system as a partner in managing their health. This index had a Cronbach's alpha of .71, indicating moderate reliability consistent with its use in this type of exploratory analysis [72].

Consistent with the strong literature demonstrating the link between prevention attitudes and behaviors, we found that these attitudes were indeed predictive of mammography behaviors. In testing the construct validity of this measure, we found it to be significantly and positively correlated with both time since last mammography and intention to receive future mammography.

### Analysis

We were interested first in understanding the prevalence of the experiences and perspectives of interest in our study population, and also how these experiences and perspectives varied in different subgroups of our population. We conducted a bivariate analysis to examine relationships between our nine psychosocial characteristics of interest, and our measures of attitudes, experiences and screening index scores. In Tables 1 and 2, we report means and t-tests for continuous measures, and Chi Square statistic for categorical measures. In Table 3, we report the pairwise associations between attitudes, experiences, and screening motivation index scores, using Pearson correlation coefficients.

In Table 4, we use multivariate linear regression to examine our outcome of interest, a positive attitude towards mammography, in relation to psychosocial characteristics, perspectives and experiences. We present two multivariate models: a full model, including all independent predictors, and a final most parsimonious model, including only those variables significant at the p < .05 level, using backwards elimination. For the multivariate analysis, we standardized our continuous measures of age, years of formal schooling, CES-D score, anomie score, and societal racism score, by centering at the population mean, and dividing by the standard deviation. (Such arithmetic operations do not change relationships for main effects, but allow for the interpretation of interaction terms at values relevant in the population, such as the mean, rather than extreme values [76]). To examine modifying effects, after building the most parsimonious model of main effects, we tested whether model fit was improved by adding, one at a time, relevant two-way interaction terms of psychosocial characteristics, attitudes and experiences. We tested whether the effects of anomie, reported racism, societal racism, talking to others when experiencing discrimination, and physician race preference varied significantly by age, education level, or depressive symptoms.

**Figure 1:**
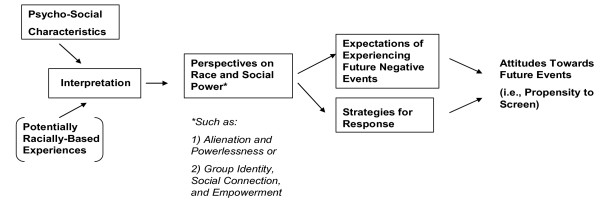
**Theoretical Model of the Pathway between Perceived Racial Discrimination and Attitudes Towards Breast Cancer Screening**. In Figure 1, persons experience events which they may or may not interpret as racially discriminatory, with the interpretation based in part on their own psychosocial characteristics. (The events are enclosed in parenthesis to denote that they are not directly observed or evaluated by others). This interpretation influences the individual's views on society, which shape both the anticipation of future experiences and the individual's planned reactions, and thus motivation to engage in health behaviors such as breast cancer screening.

We used mediational analysis [77,78] to explore further the mediating effects of worldviews and interpretations on the relationship between reported racism and screening motivation, as theorized in our model in Figure 1. To explore the relationships on the left side of the model, between reported racism and worldview, we first used simple linear regression to estimate the relationship between reported racism and screening motivation, as well as reported racism and each of two potential mediating variables: societal racism and the respondent's reported typical response when experiencing racism (talking to another versus keeping it to herself). Next, we modeled two independent variable linear regression equations, predicting screening motivation from both reported racism and these two potential mediators.

To explore relationships on the right side of the model, between world views and more proximal attitudes about medical care, we first used simple linear regression to estimate the relationship between anomie and screening motivation, as well as anomie and preference for a Black medical provider. Next, we modeled a two independent variable linear regression equation, predicting screening motivation from both anomie and preference for a Black provider.

**Figure 2:**
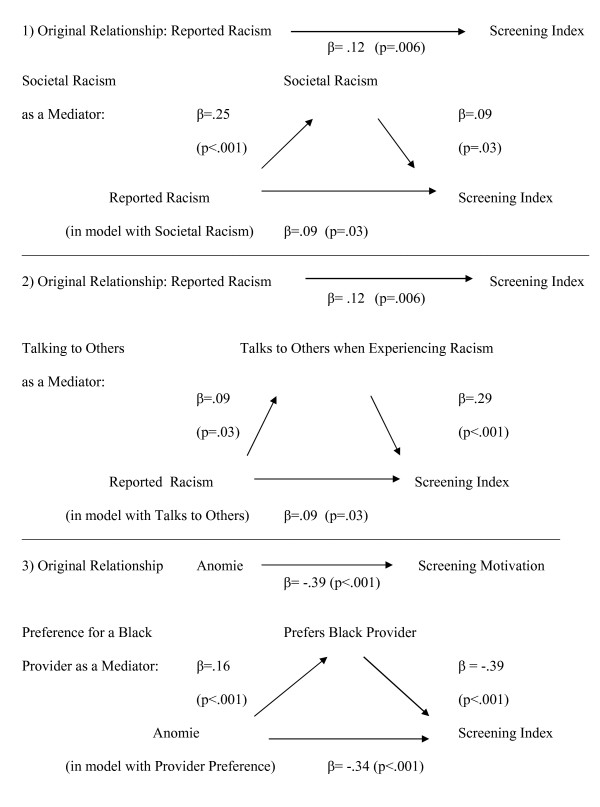
**Results of Mediational Analyses Testing Possible Pathways Between Perceived (Reported) Racism and Breast Cancer Screening Motivation**. Figure 2 depicts three different mediational analyses, testing pathways from figure 1. In analysis 1, the effect of reported racism on screening motivation is shown to be partially mediated by views on societal racism. In analysis 2, reported racism is partially mediated by the strategy of talking to others when experiencing unfair treatment. In analysis 3, the effect of anomie on screening motivation is partially mediated by preference for a Black physician.

The results of these analyses are displayed in Figure 2. SPSS statistical software [79] was used for all analyses.

## Results

### Table 1: descriptive statistics

**Table 1:** Attitudes and Experiences of Disadvantage by Respondent Characteristics

	**Perspectives on Society**		**Own Life Experiences with Racism**		
	Anomie (range: 5–20) Mean	Societal Racism (range:13–52) Mean	Reported Racism %	Talks w/Others %	Takes Action %
Total Group (n = 576)	15.5	35.1	55	86	65
**Age n**					
45–64 (356)	15.4	35.0	60 ^b^	90 ^b^	71 ^c^
65–93 (220)	15.7	35.0	46	81	56
**Yrs of School**					
< 12 (323)	16.5^c^	34.6	47^c^	79^c^	57^c^
12+ (253)	14.2	35.7	64	96	75
**Income**					
<$10,000/yr (252)	16.3^c^	34.8	51	81^b^	61
>$10,000/yr (324)	14.9	35.4	57	90	68
**Self-Rated Health**					
Fair/Poor (254)	16.1 ^c^	35.0	54	84	59^b^
Good/Excellent (322)	15.0	35.2	55	88	70
**CES-D Symptoms**					
None (283)	14.9 ^c^	34.7	52	88	64
One or More (293)	16.1	35.5	57	85	66
**Work Status**					
Working (239)	15.0^b^	35.8^a^	56	89	68
Not Working (337)	15.9	34.6	54	84	63
**Home Ownership**					
Owner (326)	15.0 ^c^	35.5	58	88	66
Renter (250)	16.2	34.6	50	84	63
**Church Attendance**					
≥ Weekly (372)	15.3^a^	34.8	57	89 ^a^	64
< Weekly (204)	15.9	35.7	50	81	66
**Locally Involved**					
Yes (282)	15.1 ^b^	35.6	63 ^c^	92 ^c^	70 ^b^
No (294)	15.9	34.6	46	81	60

a p < .05 b p < .01 c p < .001

Table 1 results illustrate both the social diversity of this population of older low income women, and also the relationship of these diverse attributes to life perspectives and experiences. Study participants ranged in age from 45 to 93, with 62% younger than 65. Years of formal schooling ranged from only 3 to over 20, with 56% having fewer than twelve years. (In Maryland and across the southern US prior to integration of schools, few African-American high schools offered a twelfth grade.) Forty-eight percent resided in households with incomes of less than $10,000 per year (compared to only 9.5% of all US households in 1999 [80]).

In terms of social spheres, all worked at some time in their lives, with 41% employed at the time of the study. Fifty-six percent lived in a home owned by themselves or a family member. Almost two-thirds attended religious services or activities once a week or more, and 49% had been involved in community affairs in the past year.

Anomie scores were fairly high overall in this group (mean = 15.5, index range 5–20), indicating a strong level of underlying hopelessness. However, as Durkheimian theory would predict, significantly lower levels of anomie were found among women with comparatively greater social, psychological, and physical resources. Lower rates of anomie were reported by those with more years of education and having more income. In addition, better physical and mental health were also related to lower rates of anomie. All four measures of social connectedness – homeownership, employment, religious and community involvement – were also significantly associated with lower rates of social alienation.

Similarly, this population in aggregate sees a significant level of societal racism against African-American women (mean = 35.1, range 13–52). However, in contrast to anomie, there was little variation by social characteristics. Women not working outside the home were less likely to view U.S. society as discriminatory against African-American women, perhaps identifying a group somewhat protected from public interactions, with less opportunity to observe societal racism.

Regarding their own experiences, 55% of respondents report perceived racial discrimination. Younger women were significantly more likely than older women to report perceived racism, as were more educated women. Reported racism was positively associated with higher levels of community involvement.

In reaction to unfair experiences, the great majority of women (86%) reported that they talk to others, rather than keeping it to themselves, while fewer (65%) reported trying to do something, rather than accepting it as a fact of life. These two strategies were more prevalent among groups with greater personal and social resources – younger women, women with more years of schooling, those with better health or higher incomes, more frequent church attendance, and community involvement.

### Table 2 – medical care experiences and perspectives

**Table 2:** Respondents' Medical Care Attitudes by Social and Psychological Characteristics

	**Race Concordance w/MD**				**Fears Research At Hospitals**	Screening Motivation*
	**Prefers**		**No Preference**			
	**Has**	**Does Not**	**Has**	**Does Not**		
	%	%	%	%	%	Mean
Total Group (n = 576)	11	21	13	55	59	31.2
**Age n**						
45–64 (356)	10	16	15	59 ^b^	57	32.4 ^c^
65–93 (220)	11	29	10	50	62	29.2
**Yrs of School**						
< 12 (323)	12	27	12	49 ^b^	63^a^	29.8^c^
12+ (253)	10	13	14	63	54	33.1
**Income**						
<$10,000/year (252)	11	26	14	49^a^	58	29.7^c^
>$10,000/year (324)	10	17	13	60	61	32.4
**Self Rated Health**						
Fair/Poor (254)	12	22	11	54	61	30.4^b^
Good/Excellent (322)	10	19	15	52	57	31.9
**CES-D Symptoms**						
None (283)	9	17	14	60 ^a^	59	31.8^b^
One or More (293)	12	25	12	51	59	30.6
**Work Status**						
Working (239)	11	16	11	62 ^a^	58	32.3^c^
Not Working (337)	10	25	15	50	60	30.5
**Home Ownership**						
Owner (326)	11	19	14	56	57	31.8^b^
Renter (250)	11	23	12	54	62	30.5
**Church Attendance**						
≥ Weekly (372)	11	18	14	57	58	31.5
< Weekly (204)	11	25	12	52	61	30.7
**Locally Involved**						
Yes (282)	13	19	13	55	56	31.8^a^
No (294)	8	23	13	56	62	30.7

* Possible index scores range from 13 to 43

a p < .05, b p < .01, c p < .001

Despite their low resources, respondents mirrored national patterns for older women in that they were, for the most part, consumers of medical care: 91% reported having a regular source of care, and 78% had some form of health insurance (data not shown). In Table 2, we see that experiences related to health care varied by psychosocial characteristics.

First we examined racial concordance with current medical provider, as well as comfort level with African-American versus other race physicians. Overall, 32% of respondents agreed with the statement that they would be more comfortable with an African-American doctor. Explanatory audiotaped comments included both rejection of race preference – "A good doctor is a good doctor" – as well as cultural preferences taking precedence over race – "He does not have to be African-American, just so long as he is some kind of American." (In comparison, 53% of respondents agreed that they would feel more comfortable seeing a woman physician than a man.)

However, only 24% of respondents reported having a primary care provider who was African-American. (The remaining 76% represent 67% whose primary care providers were not African-American and 9% who reported not having one usual source of primary care). Having a black provider was more common among women who expressed greater comfort with same-race providers (34%) than among those who said they did not agree with the statement (19%), although in these cross-sectional data, we cannot assess whether comfort level preceded, and possibly influenced provider choice, or vice versa.

These patterns of comfort and actual provider race varied by respondent age, work status, income, and CES-D symptoms. Younger, better educated, higher income, employed, or less depressed women were less likely to express provider race preference than older, less educated, non-working, poorer, or more depressed women, who were especially likely to not have a black provider, but wish for one.

The data reveal evidence of mistrust of at least some of the health care institutions within their communities. Fifty-nine percent of the respondents would be concerned about receiving care from research institutions, for fear of being deceived about research involvement. The only women with significantly greater fear were the less educated. However, it is fair to say that this fear was common, as there is no subgroup category in which the majority of respondents did not express this concern.

Finally, in Table 2, we examined the average score on the motivation for screening index among subgroups of respondents (mean score = 31.2, standard deviation = 5.5). As predicted, groups with higher motivation to be screened on a regular basis for breast cancer included younger, better educated, and wealthier women, as well as those in better physical and mental health. Additionally, working women, homeowners, and those who were involved in their communities were also more motivated to be screened. Religious participation was not associated with screening motivation in the bivariate analysis, perhaps due to greater religious involvement among older women.

### Table 3. correlations between perspectives, experiences and attitudes toward screening

**Table 3:** Pearson Correlations between Perspectives, Experiences, and Screening Motivation (N = 576)

	**Perspectives**		**Experiences**		**Medical Care**		**Screening**	
	Societal Racism	Reported Racism	Talks About it	Does Something	Has AA Provider	Wants AA Provider	Fears Research	Screening Motivation
Perspectives								
Anomie	.05 (0.23)	-.04 (0.38)	-.13 (0.001)	-.17 (<0.001)	-.06 (0.18)	.16 (<0.001)	.13 (.002)	-.39 (<.001)
Societal Racism		.25 (<0.001)	.09 (0.02)	.05 (0.21)	-.05 (0.20)	.09 (0.04)	.08 (0.06)	.12 (0.004)
Experiences								
Reports Racism			.09 (0.03)	.04 (0.32)	-.02 (.66)	.04 (0.30)	.04 (0.34)	.12 (0.006)
Talks About it				.33 (<0.001)	-.05 (0.25)	-.12 (0.004)	-.06 (0.15)	.30 (<0.001)
Does Something					-.01 (0.78)	-.12 (0.005)	-.06 (0.14)	.24 (<0.001)
Medical Care								
Has AA Provider						.16 (<0.001)	.06 (0.14)	-.10 (0.02)
Wants AA Provider							.08 (0.05)	-.39 (<0.001)
Fears Research								-.16 (<0.001)

In Table 3, results indicate that these experiences and perspectives did not represent a single phenomenon, and were differentially held by subgroups within the survey population, as Tables 1 and 2 suggested. Racial awareness appears to have taken several forms in this population. Perceived powerlessness, as measured by anomie, was weakly associated with preferring an African-American physician (r = .16, p < .001), and fearing research-related victimization at large hospitals (r = .13, p < .001). However, anomie was not significantly related to either societal racism (r = .05, p = .23), or to reported perceived racism (r = -.04, p = .38). As might be anticipated, it was weakly negatively related to talking about (r = -.13, p < .001) or taking action against discrimination (r = -.17, p < .001).

Views on societal racism against African-American women were modestly correlated with reports of one's own experiences of racism (r = .25, p < .001), and to a lesser degree with talking about racism (r = .09, p < .02), wanting an African-American provider (r = .09, p = .04), and fearing research (r = .08, p = .06). Interestingly, reporting perceived racism was not related to provider preference or research fears; rather it was the general coping strategies a woman says she typically takes that predicts her views on medical care. Those who talk to others or take action when experiencing racism were less likely to express preference for African-American providers (r = -.12, p < .01).

The last column in table 3 describes the correlations between the eight measures of perspectives and experiences and scores on the screening motivation index. On a bivariate level, anomie and greater comfort with an African-American provider have moderately negative correlation with screening motivation (r = -.39, p < .001); more modest, but still statistically significant negative correlations are seen between fear of research (r = -.16, p < .001) and currently having an African-American provider (r= -.10, p < .02). Positive correlations with screening motivation are seen with societal racism (r = .12, p = .004), reported perceived racism (r = .12, p < .006), talking about (r = .30, p < .001) and doing something about racism (r = .24, p < .001).

### Table 4. multivariate model of motivation for screening

**Table 4:** Multivariate Regression Analysis. Social and Attitude Factors Predicting Score on Index of Positive Attitudes towards Screening (n = 576)

	Full Model Model R^2 ^.43			Final Model Model R^2 ^.45		
Variables	B	Std Error	p val	B	Std Error	p val
Constant	31.16	.79	<.001	31.34	.56	.001
Psychosocial Variables						
Age	-1.32	-1.21	<.001	-1.25	.19	.001
Years of Education	.47	.21	.03	.35	.22	.10
Employed Now (0,1)	-.57	.41	.17			
Home Owner (0,1)	-.08	.39	.84			
Inc <$10,000/yr (0,1)	-.81	.40	.04			
Fair/Poor Health (0,1)	-.07	.38	.86			
Weekly Church (0,1)	-.03	.39	.94			
CES-D Index Score	-.78	.19	<.001	-2.05	.38	.001
Community Involvement (0,1)	-.11	.37	.77			
General Attitudes and Experiences						
Anomie Index	-1.31	.20	<.001	-1.36	.19	.001
Societal Racism Index	.64	.19	.001	.65	.18	.001
Self-Reported Racism (0,1)	.44	.38	.25			
Talks About Discrimination (0,1)	1.78	.57	.002	1.72	.54	.001
Does Something re: Discrimination (0,1)	.59	.40	.15			
Medical Setting-Specific						
Attitudes and Experiences						
Racial Concordance						
Neither Has Nor Prefers	ref			ref		
Does Not Have/Prefers	-2.50	.48	<.001	-2.25	.46	<.001
Has/Does Not Prefer	-.10	.55	.85	ref		
Has/Prefers	-3.97	.60	<.001	-4.11	.57	<.001
Fears Research (0,1)	-0.99	.37	.007	-.94	.37	.008
Educ*Does Not Have AA MD, Prefers				1.11	.41	.007
CES-D * Talks About Discrimination				1.81	.42	<.001

Note: Continuous variables are standardized by centering at the mean, and dividing by the standard deviation. Final model includes only those variables significant at the p < .05 level, using backward elimination.

In Table 4, the final model included two psychosocial factors recognized to influence screening attitudes and behaviors. Age had a strong negative effect on screening motivation, and women with higher scores on the depression index were significantly less likely to be highly motivated to receive breast cancer screening. In the final most parsimonious model, none of the other nine psychosocial variables had significant direct effects on screening motivation. However, education level was involved in a significant interaction.

Several of the measures of perspectives and experiences had significant independent influences on screening motivation. Higher scores on the index of anomie were negatively associated with screening motivation; in contrast, higher scores on the index of societal racism were positively associated with motivation to receive breast cancer screening.

Reported perceived racism in and of itself was not significantly predictive of screening motivation. However, one specific strategy, talking to others when experiencing discrimination, was positively associated with screening motivation. Trying to do something about discrimination, versus accepting it as a fact of life, was not predictive of screening motivation score.

Of the four possible categories of having an African-American medical provider, and feeling more comfortable with one, two were significantly negatively predictive of screening score. Women who agreed that they would be more comfortable with a black doctor, regardless of their current provider's race, expressed lower levels of motivation to receive screening. Finally, a significant direct effect was seen for women who expressed fear of receiving research treatments without their knowledge. Women who said they would be concerned about this were significantly less likely to be motivated to receive screening.

The first of two significant interaction terms shows that the effect of feeling greater comfort with an African-American doctor, but not having one, differed for women of different education levels. At the mean education level (11 years of school) or below, women in this category were less motivated to receive screening than those in the reference categories. However, as education level increased, this mismatch became a positive predictor of screening motivation.

A second interaction was seen between depressive symptoms and talking about discrimination experiences with someone else. There was a significant main effect of talking with someone else, meaning that women who used this strategy were more motivated to be screened than those who kept discrimination experiences to themselves. However, as a woman's reported number of depressive symptoms increased, this strategy became even more influential in predicting who was motivated to be screened and who was not. Those at greatest risk for poor screening motivation, therefore, were women with depressive symptoms who also did not talk to others when experiencing discrimination.

The final most parsimonious model using both sociodemographic and attitude measures to predict screening motivation score had an R^2 ^of .45, indicating that 45% of the variance in motivation score was explained by these eight independent variables. (In comparison, a final model of only sociodemographic influences on screening motivation had an R^2 ^of .26 (data not shown)).

### Figure 2 – mediational analyses of perceived racism, possible interpretations, and screening motivation

Results of the mediational analyses provide additional information about the bivariate correlations and the multivariate results. The original relationship, as also reported in Table 3, shows a positive relationship between reporting experiences of perceived discrimination and screening motivation (β = 0.12, p = .006). In the first mediational analysis, we test whether this relationship is mediated by views of the larger society's level of discrimination towards African-American women, measured by the Green Scale. Higher scores of societal racism are positively related to screening motivation (β = 0.09, p = .03); reported racism is also positively related to the societal racism (β = 0.25, p = .001). When both reported and societal racism are included in a model, the strength of the relationship between reported racism and screening is reduced (β = 0.09, p = .03); thus we can confirm that global views on discrimination against African-American women partially mediates the relationship between personal experiences and motivation to screen. Similarly, using the strategy of talking to others when experiencing racism partially mediates the relationship between reported racism and screening motivation, again reducing the relationship (β = 0.09, p = .03) when both variables are included in the model.

The relationship between anomie and desiring a Black provider is positive (β = .16, p < .001), and both anomie and wanting a Black provider have a strong negative effect on screening motivation (β = -0.39, p < .001). When we add provider preference to a model of screening motivation, it partially mediates the effect of anomie (β = -.34, p < .001).

## Discussion

Our goal was to identify experiential pathways through which social characteristics might predict differences in health maintenance attitudes. We can interpret our findings as showing three groups of influences on motivation to maintain one's health in partnership with the medical system: psychosocial characteristics, barriers to health engagement, and buffers against such barriers.

There are two significant social influences on screening motivation: age and educational level. The negative effect of age on these women's motivation for health maintenance was strong, and was only minimally reduced through the introduction of many important intermediate influences. Therefore we can speculate that cohort influences on learning about, and acceptance of, medical practices such as cancer screening are fundamental and powerful.

Older cohorts of women were socialized into medical care at a time when mammography and current philosophies of cancer control through secondary prevention were not as widely promulgated as they are today. Therefore, it is understandable that older women are less knowledgeable about, and less convinced of, the message of mammography use for successful breast cancer control.

We can also speculate that the active partnership model of patient behavior was less acceptable when these women were adopting their persona as patients. Especially for African-American women from lower social classes, traditional physician-patient relationship behaviors still predominate. Although we attribute these age differences to cohort effects, attitudinal changes with aging are also possible; this could be tested in a panel of women over time.

In contrast to age effects, education level, although important, worked through an interaction with other more immediate experiences, and must be considered in relation to those. The second important group of influences were barriers to health engagement. They appear to have operated at many levels – from societal to interpersonal to individual. Feelings of powerlessness are important barriers to health maintenance motivation – and there were three distinct types of powerlessness expressed by our respondents.

The first significant negative influence on screening motivation was a measure of depressive feelings. Although not a clinical assessment, these self-reported feelings clearly were a strong indicator of psychological burden among a substantial portion of our respondents. Unrecognized or undertreated depression among low resource groups such as the elderly and minorities puts these groups at risk for poor health maintenance, over and above barriers presented by age and poverty [81-83].

The other negative influence was anomie, a wider more philosophical measure of hopelessness, measuring powerlessness on a social level. Although these two measures were positively correlated, they both contributed independently to reducing a woman's motivation to maintain her health. Thus both personal and social hopelessness impeded health maintenance.

The third factor – a fear of being taken advantage of within the medical system itself through research-again had a specific independent role as a predictor of lower engagement in screening. Women who, in addition to societal and interpersonal hopelessness, also feared their vulnerability specifically within medical care settings, were more likely to score poorly in terms of health maintenance attitudes. The meaning of this result is significant in this urban setting, where much of the available care is affiliated with, or directly provided by, large academic hospitals. If urban residents seek to avoid care from providers and institutions affiliated in their minds with "research", they will find themselves facing additional limitations to good care, beyond those already presented by their poverty.

We identified several mechanisms by which respondents were buffered against these negative forces, and were more likely to report attitudes conducive to health maintenance. The first was acknowledgment of the negative experiences of African-Americans in many aspects of American society, as measured by the societal racism scale. We can interpret this as a form of race consciousness. This perspective allows women not to blame themselves for their negative experiences, but to attribute them to pervasive historical and social forces [84].

The second is the interpersonal strategy of discussing negative experiences attributed to racial discrimination, rather than keeping them to oneself. This can be thought of again as a method of personal empowerment, to seek connection to others rather than remain alone in one's experience. This talking strategy may be most important in promoting health maintenance attitudes for women who are depressed. Thus we see that women establish both direct and indirect social bonds – with women they know directly, and those they feel close to through the experience of race – and use those social bonds to maintain their health.

The second interaction term illustrates the complexity of interpreting our final set of findings – the relationship between the race of a current medical provider, and woman's self reported comfort level with African-American and non African-American physicians. The main effects for provider race and comfort levels showed that women who felt more comfortable with an African-American doctor, regardless of whether they had one currently or not, scored significantly lower on the breast cancer screening index.

At the reference level of education (11 years), the most significant main effect was seen in women who currently saw an African-American provider, and also agreed that they would be more comfortable with this race of physician. The negative effect of preference for a Black physician, when the woman did not currently see a Black physician, was less, although still statistically significant. In addition, this effect differed significantly by the woman's education level. The fewer years of formal education a woman reported, the stronger the negative effect of this preference was on screening motivation. However, as a woman's level of education increased, this negative effect was modified, and, at the highest levels of education, these women were just as likely to express views conducive to screening as the reference group of provider categories.

Therefore, the women with the lowest levels of health promoting views were poorly educated women who did not have access to an African-American physician, but would be more comfortable with one. These women scored poorly on all components of the health motivation index – on patient empowerment, on knowledge, and on confidence that they could fight cancer and win. In the absence of their own educational resources, they may feel a need to rely on a powerful health partner-someone of their own race to take responsibility for their health.

At the other end of the spectrum are well educated women who also did not have a black provider, but would have liked one. These women were empowered to care for themselves, perhaps in recognition that they could not count on non-black providers to take care of them. These women were just as likely as women without provider race preference to score well on the motivation index. Among educated women, the only group having a significantly lower score on screening motivation were women who have a black provider, and were more comfortable with one. These women may have adopted more passive roles than similarly educated women seeing non black providers, because they had greater trust in their physicians.

In order to explore for potential confounding effects in these results, we ran analyses omitting women without any regular provider. We also examined race-gender provider patterns, as well as insurance and practice type (clinic vs. solo practitioner) by physician race, with no change in findings.

Additionally, it is important to consider one variable which did not remain statistically significant and therefore was not included in the final models. Self-reported perceived discrimination was not a significant predictor of screening motivation, when anomie and societal racism perspectives were included. Therefore, we can speculate that these explanatory beliefs, indicating either isolation or group identity, may represent the translation of experiences into strategies for appraisal and response, and that these interpretations in turn have a more proximal effect on women's attitudes, and perhaps actions. We found further support for this interpretation in our mediational analyses which showed decreased significance for self-reported perceived racism in regression models when societal racism and talking to others when experiencing discrimination were added. Furthermore, we found evidence that worldviews, such as anomie, may directly influence health maintenance attitudes, but may also work through intervening variables, such as provider preference.

### Limitations and further work

This work is limited by the cross-sectional measurement of experiences and attitudes, and can only suggest causal pathways, rather than confirm them. Although it examines one specific subgroup within the U.S. population at one timepoint, many of the trends we observed in relation to the frequency of, and characteristics associated with, perceived discrimination are consistent with the existing literature, supporting the generalizability of our findings.

However, where our findings differ, it is important to consider possible explanations. For example, more of our respondents reported same-race provider preference than a national sample of African-Americans surveyed by telephone in 1999 (32% vs 22%) [59,60]. One possible explanation is suggested by the fact despite greater preference, slightly fewer of our respondents had black providers than the national sample (24% vs. 27%). Overall, this is consistent with fewer medical care options and poorer quality of care among this disadvantaged sample, compared to a national sample of African-Americans of all social groups. Furthermore, these data from one urban area may capture locally relevant issues such as ambivalence towards care provided by historically white research institutions. Finally, our face-to-face home-interviews conducted by black women interviewers, with markedly higher participation rates than the national survey (>90% vs. 49%), may have elicited greater disclosure of this sensitive issue than anonymous telephone surveys of national samples. For all these reasons, in-depth work in single populations has value in triangulating results of larger national surveys, and identifying questions for further inquiry.

How do the results from these analyses help answer the initial questions asked? The findings suggest that both personal and social disengagement are important barriers to health maintenance, and addressing both are important to promote health. However, results also suggest that, in addition to the need to address health care system issues, successful strategies may lie within disadvantaged populations themselves.

Specifically, the link between depressive and anomic feelings on one hand, and connection to others like oneself on the other, suggests that encouraging women to speak up about their negative experiences does not cause them to turn away from the majority system, but may in fact help them to use it. Moreover, these cross-sectional findings do not support the theory that perceived racism, whether in one's own lived experience or in society at large, is associated with depression, powerlessness, or reluctance to use medical care. Quite the opposite, perceptions of racism may, as others have observed [84], provide explanatory models for disadvantaged groups that can identify mechanisms with which to counter negative experiences.

Structuralist theory offers one lens through which to interpret these findings. At one extreme, we see that women who were truly alienated from society were poorly motivated to screen. The health consequences of social disengagement for both the individual person as well as societies and social groups has been shown across a wide range of health outcomes, most famously in Durkheim's conceptualization of anomic suicide. Our findings extend this effect of anomie to increasing the likelihood that a woman will engage in a less dramatic form of self-injurious behavior, if we interpret her attitudes to indicate she is less likely to use secondary prevention to protect herself against a future health risk. This finding suggests that early detection of cancer cannot be promoted in disadvantaged social groups without considering how to mediate the impact of social alienation on health attitudes.

On the other hand, we see that women who failed to recognize racial inequalities in American society were also at increased risk for attitudes associated with poor screening. In considering the 'curvilinear' effects of the tension between individuals and society, Durkheim proposed that there was a danger of altruistic as well as anomic suicide. Extending Durkheim's example of military officers whose 'passive habit of obedience (leads them) to undervalue their own lives,' [[85], p. 134–135] altruistic suicide is committed by those who are so overregulated by society and so enmeshed in their social roles that they cannot refuse to fulfill any negative aspects of those roles. We can speculate that African-American women who see no racism in American society, as well as those who do not speak out against injustices in their lives, are similarly sacrificing their own well-being in support of the social order. This may explain why a middle ground, including some level of mistrust, was associated with the healthiest attitudes among these African-American women.

Providers, especially minority providers, must understand the multiple levels of influence that their own race may have on their disadvantaged patients. Greater comfort in the patient-physician relationship may result not only because of positive bonds between patient and provider, but also because of fears or negative experiences involving other race providers [60,62]. Our data suggest that persons most desiring same race providers and not having one are the most disadvantaged members of these communities. To the extent that those fears further restrict these patients from using care or protecting their health, they must be addressed. Again, these findings suggest that the solution does not lie in persuading these patients that the majority system is trustworthy or that individual providers can shield them from disadvantage and discrimination. It may lie instead in empowering these women, through connection to others like themselves, to claim equal "candidacy" in the health care system, make informed decisions for themselves and take control of their own health.

## Appendix: Index of Positive Motivation For Screening

### Domain 1: Rejects Fatalistic Explanations of Cancer, from a list of 15 Possible Causes

Now I am going to read you a list of some of the reasons that people might use to explain who gets breast cancer and who does not. For each reason, please thing about how much this might explain whether a woman gets breast cancer. Please tell me if you think it has a BIG EFFECT, SOME EFFECT, NOT MUCH EFFECT or NO EFFECT AT ALL on whether or not a woman gets breast cancer.

1) Contagious elements, such as a virus, and having direct contact with someone who has breast cancer.

2) Having the kind of personality that causes cancer.

3) Punishment for something a person has done wrong in her life.

### Domain 2: Acceptance of Cancer as A Treatable Disease

4) If I had breast cancer, I would rather not know about it.

5) Cancer would be the worst disease I can imagine having.

### Domain 3: Knowledge of Breast Cancer Control

6) "In your own words, can you tell me what a mammogram is, and what the purpose of it is?"

Defines Mammography as an X-Ray of the Breast: Yes/No

Defines Purpose as a Test for Breast Cancer: Yes/No

7) In most cases, by the time a doctor can see a breast cancer the size of a pin head on a mammogram, ...what is the chance of it already having spread to another part of her body? Is it not at likely, somewhat likely, very likely, or almost certain to have spread?

8) After a woman has had 2 or 3 negative mammograms, it is not necessary to have any more.

### Domain 4: Role of Patient as Co-Equal with Provider in Cancer Control

9) If you trust your doctor, you do not need to ask for any tests. He or she will give them to you when you need them.

10) Regarding my health, I can only do what my doctor tells me to do.

11) Women can tell if they have breast cancer without going to the doctor for tests.

SCORING KEY: Items 1–3 highest score to "No effect at all". Items 4,5, 8–11: Likert Scale (strongly agree, agree a little, disagree a little, strongly disagree), highest score to "strongly disagree." Item 7: highest score to "not likely at all"

## Competing interests

The author(s) declare that they have no competing interests.

## Authors' contributions

ACK was the principal investigator for the parent study for these data, designed and conducted the statistical analysis, and drafted the paper. KCS contributed to the theoretical framework for the analysis, and contributed substantially to the final version of the paper. SSM contributed to the interpretation of the results, and to the final version of the paper. HSJ advised the authors on conducting and interpreting the mediational analyses, as well as contributing to the final version of the paper. All authors read and approved the final manuscript.
